# Characteristics of lipid metabolism including serum apolipoprotein M levels in patients with primary nephrotic syndrome

**DOI:** 10.1186/s12944-017-0556-9

**Published:** 2017-09-06

**Authors:** Lagu He, Pengfei Wu, Li Tan, Bai Le, Wenhan Du, Ting Shen, Jiali Wu, Zheyi Xiang, Min Hu

**Affiliations:** 1 Department of Laboratory Medicine, The Second Xiangya Hospital, Central South University, Changsha, Hunan 410011 China; 2 Department of Spine Surgery, The Second Xiangya Hospital, Central South University, Changsha, Hunan 410011 China

**Keywords:** Apolipoprotein M,PNS, HDL, Lipids, Hyperlipidaemia

## Abstract

**Background:**

Apolipoprotein M (apoM) is a 26-kD apolipoprotein that is mainly expressed in specific cell types, such as human liver parenchymal cells and kidney proximal renal tubular epithelial cells. ApoM can regulate the formation of pre-β-HDL and the reverse cholesterol transport and thus plays an important role in the metabolism of lipids and lipoproteins, meaning that it can affect the development of lipid metabolism disorders. Significantly elevated serum apoM levels are detected in patients with hyperlipidemia. However, few studies have shown how apoM is expressed in primary nephrotic syndrome (PNS), which is often accompanied with hyperlipidemia, and the underlying mechanism is poorly understood. This study was aimed at examining the apoM levels in patients with PNS and at determining the effects of PNS on serum apoM levels in these patients.

**Methods:**

This study included patients with hyperlipidemia (*n* = 37), the PNS with hyperlipidemia group (*n* = 62), PNS without hyperlipidemia group (*n* = 33), and healthy controls (*n* = 73). The age and body–mass index (BMI) matched among the groups of participants. Their serum apoM concentrations were measured by an enzyme-linked immunosorbent assay. Serum levels of conventional lipids and renal function indices were assessed using an automatic biochemical analyzer. The data were analyzed by means of Pearson’s correlation coefficient (continuous variables) or Student’s *t* test (mean differences).

**Results:**

The average serum apoM concentrations were higher in the hyperlipidemia group (61.1 ± 23.2 mg/L, *P* = 0.004) than in the healthy controls (31.6 ± 18.92 mg/L). The serum apoM concentrations were lower in the PNS with hyperlipidemia group (25.1 ± 16.31 mg/L, *P* = 0.007) and in the PNS without hyperlipidemia group (21.00 ± 17.62 mg/L, *P* = 0.003) than in the healthy controls. The serum apoM concentrations in the PNS with hyperlipidemia group did not differ significantly from those in the PNS without hyperlipidemia group (*P* = 0.083). Moreover, serum apoM levels positively correlated with serum high-density lipoprotein cholesterol (HDL-C) and apoA1 levels and negatively correlated with proteinuria in PNS patients (*r = 0.458, P* = 0.003; *r = 0.254, P = 0.022; r = −0.414, P = 0.028*).

**Conclusion:**

Serum apoM concentrations are higher in patients with hyperlipidemia than in healthy controls. Low serum apoM levels in patients with PNS are likely caused by PNS.

## Background

Primary nephrotic syndrome (PNS) is a common progressive kidney disease occurring in childhood and characterized by a selective change in the glomerular capillary wall; this change prevents the wall from limiting the loss of protein with urine [1]. Nephrotic syndrome is characterized by substantial proteinuria, hypoproteinemia, edema, and hyperlipidemia (HLP), and a group of symptoms featured in metabolic syndrome. Hyperlipidemia is often accompanied with complex dyslipidemia, such as elevated very low-density lipoprotein cholesterol (VLDL-C) and LDL-C levels and low high-density lipoprotein cholesterol (HDL-C) levels, which are associated with serum apolipoprotein M (apoM) [2].

ApoM is a 26-kD apolipoprotein that belongs to the lipocalin protein family [3]. ApoM is mainly expressed in specific cell types, such as human liver parenchymal cells and kidney proximal renal tubular epithelial cells [4]. Studies have revealed that apoM can regulate the formation of pre-β-HDL and the reverse cholesterol transport, thus playing an important role in the metabolism of lipids and lipoproteins, indicating that it can affect the development of lipid metabolic disorders, such as coronary artery disease, nephrotic syndrome, and liver disease [5]. The mechanism of nephritic HLP is complicated. The prevailing view is that the liver synthesis of lipids and apolipoproteins is increased, and the clearance rates of chylomicrons and VLDL are reduced [6]. Hyperlipidemic mice with defective LDL receptor have a high plasma level of triglycerides (TGs) and serum apoM [7]. Therefore, we hypothesized that the level of apoM may be associated with hyperlipidemia. Researchers have found that there are different characteristics of lipid metabolism at different levels of urinary protein excretion (UPE) [8]. HLP, the main complication of PNS, is thought to be involved in cardiovascular disease (CVD) and in progressive glomerular damage leading to renal failure [9, 10]. ApoM is mainly expressed in human liver parenchymal cells and kidney proximal renal tubular epithelial cells. Therefore, the specific effect of hyperlipidemia on serum apoM in patients with PNS should be studied to confirm whether the concentration of apoM is associated with hyperlipidemia or PNS. The aim of this study was to measure the apoM levels in patients with hyperlipidemia, patients with PNS and hyperlipidemia, and patients with PNS but without hyperlipidemia by an enzyme-linked immunosorbent assay (ELISA). This study was also aimed at determining the effects of serum apoM in patients with PNS.

## Methods

### Subjects

This was a case-control study of PNS patients conducted at the Second Xiangya Hospital (Changsha, Hunan, China) between July 2016 and June 2017. The diagnosis of PNS was based on each patient’s clinical history, physical examinations according to the Global Initiative. The diagnostic criteria were (1) proteinuria >50 mg/(kg⋅d), (2) plasma albumin < 25 g /l, (3) edema, (4) HLP. (1),(2) are necessary. Hyperlipidemia was defined according to the Adult Treatment Panel III of the National Cholesterol Education Program (serum total [TGs] ≥ 220 mg/dL [2.49 mmol/L] or serum total cholesterol [THOL] ≥ 240 mg/dL [6.24 mmol/L]) [11]. The exclusion criteria were (1) diabetes, (2) pregnancy, (3) thyroid disease, (4) cancer, (5) immunological disease, (6) congestive heart failure, (7) another renal disease, (8) metabolic acidosis, or (9) venous or arterial thrombosis. Hyperlipidemia patients who used lipid-lowering drugs and PNS patients who received steroid therapy were also excluded. The control group included 73 nonallergic volunteers without renal disease and 37 hyperlipidemia controls without other diseases who underwent physical examination in our hospital. The main clinical and biochemical characteristics of the four groups in this study are shown in Table 1.

**Table 1:** Clinical and biochemical characteristics of the study subjects including apoM

	Healthy controls	HLP	PNS + HLP	PNS-HLP
Subjects, n	73	37	62	33
Demographics				
Age, years	40 ± 14	42 ± 8	39 ± 13	39 ± 14
(male/female)	40/32	23/14	36/26	20/13
Body–mass index, kg/m^2^	21.4 ± 1.81	22.5 ± 2.40	22.3 ± 2.0	22.2 ± 2.0
Lipid profile:				
Triglycerides, mmol/L	0.86 ± 0.38	3.46 ± 1.90^aa^	2.54 ± 1.57^aacc^	1.86 ± 1.18^aabb^
Total cholesterol, mmol/L	4.19 ± 0.56	5.52 ± 0.90^aa^	8.54 ± 2.60^aacc^	5.47 ± 2.62^aabb^
HDL-C, mmol/L	1.59 ± 0.48^a^	1.12 ± 0.31^aa^	1.40 ± 0.25^ac^	1.35 ± 0.45^aa^
LDL-C, mmol/L	2.58 ± 0.49	3.68 ± 0.82^aa^	6.67 ± 2.50^aacc^	3.73 ± 2.28^aabb^
Apolipoprotein A, g/L	1.45 ± 0.37	1.50 ± 0.44	1.27 ± 0.20 ^c^	1.33 ± 0.19
Apolipoprotein B, g/L	0.79 ± 0.16	1.13 ± 0.22^aa^	1.85 ± 0.53^aa^	1.14 ± 0.54^aabb^
Apolipoprotein M, mg/L	31.6 ± 18.92	61.1 ± 23.21^aa^	25.1 ± 16.31^aacc^	21.0 ± 17.62^aa^
Lipoprotein(a), mg/L	184.5 ± 223.1	108 ± 116	740 ± 446.27^aacc^	433 ± 384.7^aacc^
UA (g/L)	281 ± 68.01	343 ± 68.02^aa^	346 ± 105.42^aa^	363 ± 113.32^aa^
BUN (μmol/L)	4.78 ± 1.06	5.24 ± 1.15^a^	7.27 ± 4.48^aacc^	7.89 ± 6.64^aa^
CRE (μmol/L)	59.1 ± 18.02	68.7 ± 13.81^aa^	73.8 ± 42.51^aa^	80.0 ± 61.01^aa^
CysC (mg/ L)	0.77 ± 0.13	0.82 ± 0.12^a^	1.19 ± 0.40^aacc^	1.33 ± 0.85^aa^
NGAL (ng/mL)	68.2 ± 27.01	85.0 ± 32.02^aa^	143.2 ± 76.05^aacc^	17.1, ±95.02^aa^
Proteinuria (g/d)			5.74 ± 4.50	4.16 ± 3.50^a^

Data are mean ± SD. *N* number, *HLP* HLP patients,PNS + HLP: PNS with hypoproteinemia, *PNS-HLP* PNS without hypoproteinemia, *HDL-C* high-density lipoprotein cholesterol, *LDL-C* low-density lipoprotein cholesterol

*P* value comparisons among four groups by one-way ANOVA

^a^vs. Healthy control group

^b^vs. PNS with hyperlipidemia group

^c^vs. Hyperlipidemia group

^a^ *P* < 0.05, ^aa^ *P* < 0.01

^b^ *P* < 0.05, ^bb^ *P* < 0.01

^c^ *P* < 0.05, ^cc^ *P* < 0.01

### Blood sampling

After an overnight fast and at least 20 min of rest, blood samples were collected from each subject. Serum was obtained by centrifugation at 3500 rpm for 5 min, and aliquots were stored at −80°C.

### ELISA for apoM quantification

Serum apoM levels were measured by a sandwich ELISA (Yuan Tai Bio Inc., Changsha, Hunan, The People’s vhRepublic of China). Optical density (OD) was measured at 450 nm (with a background reading at 620 nm), using an ELX-800 absorbance reader (BioTek Instruments, Inc., Winooski, VT, USA). The concentration of apoM (in mg/L) in each sample was determined using a standard curve.

### Assays of lipoproteins and renal-function indices

Levels of serum TGs, THOL, HDL cholesterol (HDL-C), LDL cholesterol (LDL-C), apolipoprotein A1 (apoA1), apolipoprotein B (apoB), lipoprotein a (Lpa), Nrinary neutrophil gelatinase-associated lipocalin (NGAL), and other renal injury biomarkers (cystatin-C [CysC], creatinine [CRE], blood urea nitrogen [BUN], and uric acid [UA]) were measured on an ARCHITECT c8000 System (Abbott Laboratories, Irving, TX, USA).

### Statistical analysis

Continuous variables are presented as mean ± standard deviation. Relations between continuous variables were tested by means of Pearson’s correlation coefficient. Categorical variables were expressed as percentages. Overall comparisons were made by one-way analysis of variance (ANOVA), and multiple comparison between the two groups was based on the LSD-t test. Differences in percentages of variables were analyzed by the χ^2^ test. The relations between an index and apoM were examined by Pearson’s linear regression analysis. Statistical analyses were conducted in the SPSS 20.0 software (SPSS Statistics, Inc., Chicago, IL, USA) or GraphPad Prism 5.0 (GraphPad Software, La Jolla, CA, USA). Data with two-tailed *P* values <0.05 were considered statistically significant.

All the subjects provided signed informed consent, and the study protocol was approved by the Second Xiangya Hospital Investigational Review Board.

## Results

### Patients’ characteristics and serum variables

**Fig. 1:**
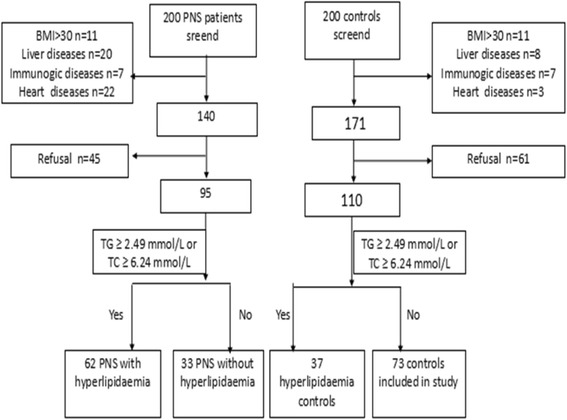
The flowchart of the study

**Fig. 2:**
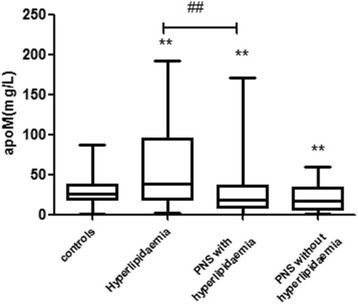
Serum apolipoprotein M (apoM) concentration is decreased in PNS patients but elevated in the hyperlipidemia group. Serum concentrations of apoM in PNS patients, grouped according to the Global Initiative for PNS, the Adult Treatment Panel III of the National Cholesterol Education Program, and in healthy controls. ^*^ *P* < 0.05, ^**^P < 0.01 as compared to controls. ^#^P < 0.05 a significant difference between the two groups indicated by the horizontal line

Figure 1 describes the patient selection flowchart for the 200 PNS patients screened for participation in this study; 60 did not meet the eligibility criteria delineated in the Methods section, and 45 refused to participate; therefore, 95 patients were finally analyzed. Additionally, 200 controls were screened for participation; 29 failed the eligibility criteria and 61 refused to participate. Accordingly, 110 control subjects were enrolled. The serum apoM concentrations were determined in patients with hyperlipidemia, PNS without hyperlipidemia, PNS with hyperlipidemia and healthy controls. No difference was observed among the four groups in gender (*P* = 0.724), age (*P* = 0.653), or BMI (*P* = 0.945; Table 1). Serum apoM levels in patients with hyperlipidemia were 61.1 ± 23.21 mg/L, i.e., much higher than those in the control group (31.6 ± 18.92 mg/L; *P* = 0.004; Fig. 2). However, the serum apoM concentrations were lower in the PNS without hyperlipidemia group (21.0 ± 17.62 mg/L,*P* = 0.003) and PNS with hyperlipidemia group (25.1 ± 16.31 mg/L,*P* = 0.007) than in the healthy control group (31.6 ± 18.92 mg/L). The serum apoM concentrations were lower in the PNS with hyperlipidemia group (25.1 ± 16.31 mg/L) than in the hyperlipidemia group (61.1 ± 23.21 mg/L; *P* = 0.001). Meanwhile, there was no significant difference between the PNS with hyperlipidemia group and PNS without hyperlipidemia group (*P* = 0.083).

### Routine serum lipid levels in the four groups

**Fig. 3:**
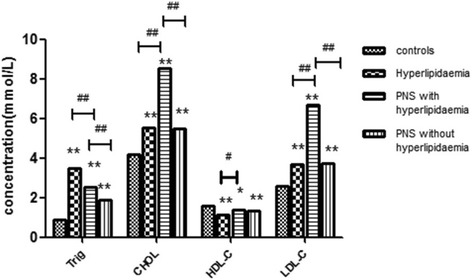
Patients with primary nephrotic syndrome (PNS) show elevated serum triglyceride (TG) levels, total cholesterol (THOL), high-density lipoprotein cholesterol (HDL-C), and low-density lipoprotein cholesterol (LDL-C). Serum concentrations of routine serum lipid levels in PNS patients, grouped according to the Global Initiative for PNS, the Adult Treatment Panel III of the National Cholesterol Education Program, and in healthy controls. *P < 0.05, **P < 0.01 as compared to the control subjects. ^##^P < 0.01 a significant difference between the two groups indicated by the horizontal line

As shown in Fig. 3, compared with the healthy controls, hyperlipidemia patients had significantly higher serum THOL, TG, and LDL-C concentrations and lower HDL-C levels. Meanwhile, the PNS with hyperlipidemia group had higher THOL, TG, and LDL-C levels as compared with the hyperlipidemia group. However, serum TG and HDL-C levels were lower in the PNS with hyperlipidemia group. When all the patients were compared with healthy children, the serum concentrations of THOL, TG, and LDL-C were higher in the PNS group than in the control group (*P* = 0.007, *P* = 0.008, *P* = 0.004).

### Renal function indices in the four groups

**Fig. 4:**
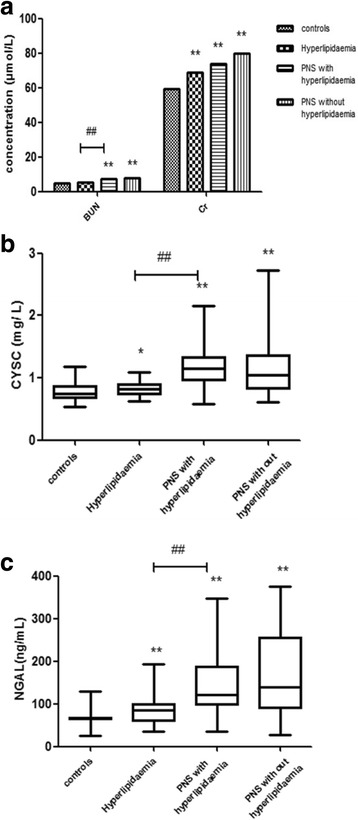
The graphs above showing the renal function indices. **a** PNS patients have elevated BUN and CRE levels; (**b**) The PNS and hyperlipidemia groups shows elevated CysC as compared with the healthy controls; the PNS groups had significantly higher serum CysC levels as compared with the hyperlipidemia group; (**c**) the PNS and hyperlipidemia groups shows elevated NGAL concentrations as compared with the healthy controls; the PNS groups show significantly higher serum NGAL levels as compared with the hyperlipidemia group. Serum levels of renal function indices in PNS patients grouped according to the Global Initiative for PNS, the Adult Treatment Panel III of the National Cholesterol Education Program, and in healthy controls. *P < 0.05, **P < 0.01 as compared to the control subjects. ^##^P < 0.01 a significant difference between the two groups indicated by the horizontal line

PNS patients had significantly higher (*P* = 0.009, *P* = 0.005 for each) serum UA and BUN concentrations than did healthy controls (Fig. 4a). However, there was no difference in UA and BUN levels between the PNS patients and hyperlipidemia patients. Moreover, the serum CysC levels of the PNS patients and hyperlipidemia patients were significantly higher than of the control group (*P* = 0.024, *P* = 0.017, respectively)(Fig. 4b). Meanwhile, similar NGAL levels were observed among the four groups (Fig. 4c). Moreover, compared with the hyperlipidemia group, the PNS with hyperlipidemia group showed significantly higher serum NGAL and CysC levels (*P* = 0.001, *P* = 0.003, respectively). There was no difference in serum NGAL and CysC levels between the PNS with hyperlipidemia group and the PNS without hyperlipidemia group(*P* = 0.294, *P* = 0.085, respectively).

### The association of serum apoM with clinical and lipid variables

**Fig. 5:**
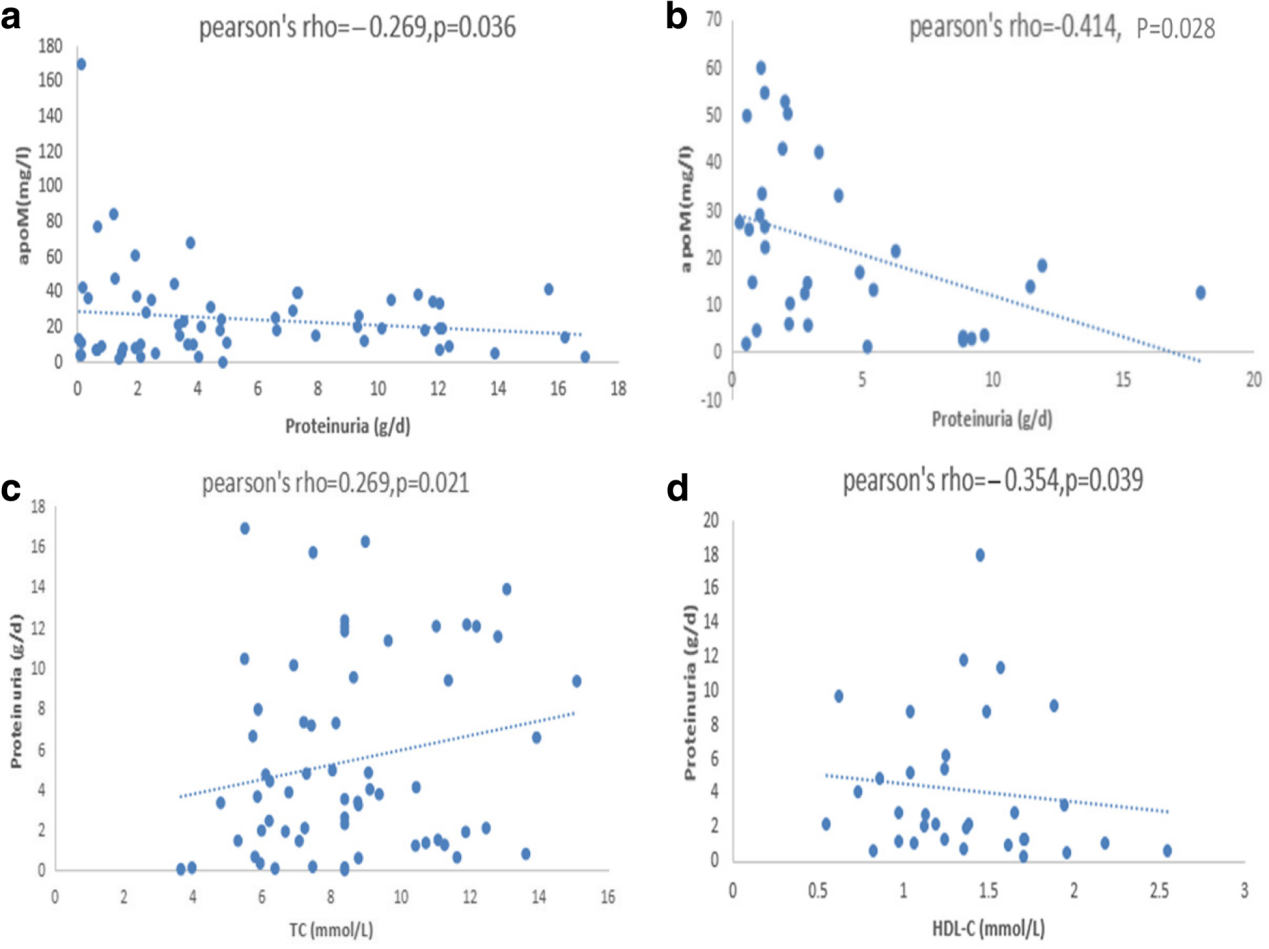
**a, b** Correlation between serum levels of apoM levels and proteinuria. **c, d** Correlation between serum levels of proteinuria and routine serum lipids. There was a negative correlation between proteinuria and serum apoM levels: (**a**) the PNS with hyperlipidemia group (r = −0.269; P < 0.05), (**b**) the PNS without hyperlipidemia group (r = −0.414; P < 0.05), (**c**) the PNS with hyperlipidemia group (*r* = 0.269; P < 0.05), (**d**) the PNS without hyperlipidemia group (r = −0.354; P < 0.05)

As shown in Table 2, the serum apoM concentration positively correlated with THOL, HDL-C, and apoA1 in the hyperlipidemia group (*r = 0.393, P* = 0.016; *r = 0.363, P* = 0.038; and *r = 0.334, P* = 0.004, respectively); we found that apoM positively correlated with HDL-C and apoA1 in the patients with PNS and hyperlipidemia (*r = 0.231, P* = 0.045; and *r = 0.324, P* = 0.019, respectively). Besides, apoM positively correlated with HDL-C in the healthy controls (*r = 0.282, P* = 0.014). The serum apoM concentration in the PNS without hyperlipidemia group also positively correlated with HDL-C, LDL-C, apoA1, and apoB (*r = 0.458, P* = 0.003; *r = 0.423, P* = 0.017; *r = 0.254, P* = 0.022; and *r = 0.427, P* = 0.036, respectively). Among the renal variables, a negative correlation was detected between proteinuria and apoM in the PNS with hyperlipidemia group and PNS without hyperlipidemia group (*r = −0.269, P* = 0.036, and *r = −0.414, P* = 0.028, respectively; Fig. 5a, b). We also found that proteinuria positively correlated with THOL in the PNS with hyperlipidemia group (*r = 0.269, P* = 0.021) and negatively correlated with HDL-C in the PNS without hyperlipidemia group (*r = −0.354, P* = 0.039; Fig. 5c, d). Besides, apoM negatively correlated with NGAL in the PNS with hyperlipidemia group (*r = −2.56, P* = 0.031) although such an association was not observed in the other three groups. Moreover, the serum apoM concentration negatively correlated with CRE in healthy controls alone (*r = −0.303, P* = 0.004). ApoM did not correlate with age, gender, Lpa, TGs, UA, BUN, or CysC.

**Table 2:** Correlation between serum lipid profiles and apoM

	Healthy controls	HLP	PNS + HLP	PNS-HLP
gender	0.196	0.206	0.085	0.045
age	0.034	−0.267	0.043	0.043
TGs (mmol/L)	−0.126	0.111	−0.124	0.260
THOL (mmol/L)	0.022	0.393^*^	−0.194	0.201
HDL-C (mmol/L)	0.282^*^	0.363^*^	0.231^*^	0.458^**^
LDL-C (mmol/L)	−0.165	−0.01	−0.197	0.423^*^
apoA1 (g/L)	−0.154	0.334^**^	0.324^*^	0.254^*^
apoB (g/L)	−0.07	0.079	−0.182	0.427^*^
Lpa (mg/L)	0.107	0.077	−0.116	0.135
UA (g/L)	−0.108	−0.223	−0.003	0.066
BUN (μmol/L)	−0.072	0.168	−0.007	0.058
CRE (μmol/L)	−0.303^**^	−0.092	0.153	0.122
CysC (mg/ L)	−0.202	−0.078	0.113	0.166
NGAL (ng/mL)	−0.042	0.099	−0.256^*^	0.036
Proteinuria (g/d)			−0.269^*^	−0.414^*^

Univariate correlation coefficients (Pearson’s r) for associations of serum apoM with clinical variables and serum parameters

Groups: hyperlipidemia, PNS without hyperlipidemia, PNS with hyperlipidemia, and healthy controls. The data indicate Pearson’s r

^*^ *P* < 0.05; ^**^ *P* < 0.01

## Discussion

Our study mainly revealed the characteristics of lipids metabolism including apoM levels in patients with PNS, and we screened these data for associations between serum apoM levels and proteinuria. The concentration of apoM in patients with hyperlipidemia was higher than that in healthy controls, and apoM concentrations in PNS patients were lower than in healthy controls. These observations suggest that hyperlipidemia increases serum apoM levels. Thus, we can theorize that low serum apoM concentrations in PNS patients were not triggered by hyperlipidemia. Instead, low serum apoM levels were likely caused by PNS. These results are similar to Zhang’s finding that the plasma apoM concentrations of hyperlipidemia patients are higher than those of healthy controls. Diabetes is the possible reason for low plasma apoM levels in patients with type 2 diabetes mellitus, but are not induced by hyperlipidemia [12]. In the present study, PNS patients showed lower serum concentrations of HDL-C, apoM, and apoA1 than did the control subjects. However, these patients had higher serum levels of THOL, TG, LDL, and Lpa. These results are similar to the data from Shearer G.C. et al. [13]. ApoM accounts for <2% of LDL and ~5% of HDL-C and primarily interacts with HDL-C. The apoM complex also contains related apolipoproteins: apoA1 and apoB [14]. Thus, the serum apoM levels here were associated with those of apoA1 and HDL-C. ApoM-HDL interactions promote pre-β-HDL formation [15] and increase cholesterol efflux from foam cells [16]. In our study, the decreased concentrations of apoM in the PNS groups may be partly caused by the reduction of HDL. However, it is interesting in one study that the level of HDL-C in patients with coronary artery disease was found to be significantly lower than that in the control group, but there was no significant difference in apoM levels between the two groups [17]. In our study, The level of apoM was increased in HLP patients, However, the HDL-C level was significantly decreased, indicating that serum apoM levels were associated with other factors such as LDL-C, THOL, and apoA1 [18]. These findings are in agreement with data from Kurano et al., which indicate that serum apoM levels are reduced in mice with overexpression of LDL receptors [19]. Meanwhile, Christoffersen found that mice with defective LDL receptor whose plasma TG levels are elevated have significantly higher serum apoM concentration, and most apoM is associated with LDL-C/CM particles, rather than HDL-C. Christoffersen et al. also found that apoM is mainly present in plasma HDL-C and shows a negative correlation with the HDL-C concentration in individuals with low HDL-C levels due to lecithin-cholesterol acyltransferase deficiency [20]. The possible explanation for this result and the relation between hyperlipidemia and serum apoM levels may be that apoM can be rapidly exchanged between VLDL-C/LDL-C and HDL-C particles in vivo. This situation suggests that apoM in the VLDL/LDL pool is replenished from the HDL pool in hyperlipidemia patients with high serum concentrations of VLDL/LDL. Primary nephrotic syndrome is also an immune-related disease, therefore, we suspect that the decrease of apoM in PNS patients may also correlate with the elevation of immune complex. The specific mechanism needs further study.

A negative correlation was detected between proteinuria and apoM, and a positive correlation was observed between proteinuria and THOL in the PNS with hyperlipidemia group. Proteinuria negatively correlated with HDL in the PNS with hyperlipidemia group in our study. The clinical manifestations of PNS include hypoproteinemia, proteinuria, edema, and HLP. Among them, proteinuria is the most important pathophysiological change and is a diagnostic criterion of PNS, which is classified by the severity of glomerular injury. First, owing to possible endothelial injury, proteinuria has been associated with elevated serum levels of proinflammatory markers and cytokines [21]. These proinflammatory cytokines can stimulate the expression and function of the liver LDL receptor gene, leading to hypercholesterolemia [22]. Second, proteinuria, a major risk factor of HLP, has been reported to be associated with fasting hyperinsulinemia [23]. In our study, the negative correlation between apoM and proteinuria in PNS patients may indicate that reduced apoM levels may reflect the severity of disease in PNS patients. Hyperlipidemia is often associated with a decrease in serum HDL-C with elevated THOL and LDL-C, which is associated with apoM [24, 25]. The possible mechanism underlying these findings may be the following: those factors, such as impaired renal function, also contribute to dyslipidemia. Our study assessed the serum apoM levels in patients with hyperlipidemia and revealed that patients with hyperlipidemia had significantly higher apoM levels than healthy controls did. In addition, both the serum apoA1 and apoM levels were higher than those in healthy controls. Moreover, there was no statistically significant difference in serum apoA1 and apoM levels between the PNS without hyperlipidemia group and PNS with hyperlipidemia group. Faber et al. have found that the plasma levels of apoM decrease to 33% of the normal value in apoA1-deficient mice [26]. Meanwhile, apoA1 and apoM mainly exist in HDL-C [27], and apoM is connected with apoA1 via metabolism. Suresh C.P. et al. have found that a lowered level of apoA1 in urine is suggestive of steroid-resistant nephrotic syndrome (SRNS) [28]. These results may also mean that apoM can predict the severity of nephrotic syndrome.

Our study showed that serum apoM negatively correlates with proteinuria in patients with PNS and provides important information for treatment selection. Future studies will shed light on the pathogenicity of these proteins for glomerular capillary integrity, and the serum levels of these proteins may serve as metabolic markers to guide the treatment of nephrotic syndrome. Because the sample size of PNS patients in this study is small, whether the serum apoM level correlates negatively with the severity of PNS is open to discussion.

## Conclusion

Patients with PNS have diverse characteristics of lipid metabolism. The serum apoM concentration in patients with hyperlipidemia was found to be higher than that in healthy controls.

Serum apoM expression is likely to be inhibited in patients with PNS,despite of the existence of hyperlipidemia in certain cases .

## Abbreviations

ANOVA: Analysis of variance
apoA1: Apolipoprotein A1
apoB: Apolipoprotein B
apoM: Apolipoprotein M
HDL-C: High-density lipoprotein cholesterol
HLP: Hypoproteinemia
LDL-C: Low-density lipoprotein cholesterol
OD: Optical density
PNS: Primary nephrotic syndrome
TG: Triglyceride
THOL: Total cholesterol
UPE: Urinary protein excretion
VLDL: Very low-density lipoprotein
